# Upregulating Human Cathelicidin Antimicrobial Peptide LL-37 Expression May Prevent Severe COVID-19 Inflammatory Responses and Reduce Microthrombosis

**DOI:** 10.3389/fimmu.2022.880961

**Published:** 2022-05-12

**Authors:** Karim M. Aloul, Josefine Eilsø Nielsen, Erwin B. Defensor, Jennifer S. Lin, John A. Fortkort, Mehrdad Shamloo, Jeffrey D. Cirillo, Adrian F. Gombart, Annelise E. Barron

**Affiliations:** ^1^ Department of Bioengineering, Schools of Medicine and of Engineering, Stanford University, Stanford, CA, United States; ^2^ Department of Science and Environment, Roskilde University, Roskilde, Denmark; ^3^ Department of Neurosurgery, School of Medicine, Stanford University, Stanford, CA, United States; ^4^ Department of Microbial Pathogenesis and Immunology, Texas A&M College of Medicine, Bryan, TX, United States; ^5^ Department of Biochemistry and Biophysics, Oregon State University, Corvallis, OR, United States; ^6^ The Linus Pauling Institute, Oregon State University, Corvallis, OR, United States

**Keywords:** NET clearance, LL-37, COVID-19, SARS-CoV-2, alpha synuclein, diabetes, neutrophil extracellular trap (NET), cathelicidin

## Abstract

COVID-19 is characterized by hyperactivation by inflammatory cytokines and recruitment of macrophages, neutrophils, and other immune cells, all hallmarks of a strong inflammatory response that can lead to severe complications and multi-organ damage. Mortality in COVID-19 patients is associated with a high prevalence of neutrophil extracellular trap (NET) formation and microthrombosis that are exacerbated by hyperglycemia, diabetes, and old age. SARS-CoV-2 infection in humans and non-human primates have revealed long-term neurological consequences of COVID-19, possibly concomitant with the formation of Lewy bodies in the brain and invasion of the nervous system *via* the olfactory bulb. In this paper, we review the relevance of the human cathelicidin LL-37 in SARS-CoV-2 infections. LL-37 is an immunomodulatory, host defense peptide with direct anti-SARS-CoV-2 activity, and pleiotropic effects on the inflammatory response, neovascularization, Lewy body formation, and pancreatic islet cell function. The bioactive form of vitamin D and a number of other compounds induce LL-37 expression and one might predict its upregulation, could reduce the prevalence of severe COVID-19. We hypothesize upregulation of LL-37 will act therapeutically, facilitating efficient NET clearance by macrophages, speeding endothelial repair after inflammatory tissue damage, preventing α-synuclein aggregation, and supporting blood-glucose level stabilization by facilitating insulin release and islet β-cell neogenesis. In addition, it has been postulated that LL-37 can directly bind the S1 domain of SARS-CoV-2, mask angiotensin converting enzyme 2 (ACE2) receptors, and limit SARS-CoV-2 infection. Purposeful upregulation of LL-37 could also serve as a preventative and therapeutic strategy for SARS-CoV-2 infections.

## Introduction

The virus SARS-CoV-2 has caused more than 6 million deaths worldwide since its arrival in December of 2019 (1). SARS-CoV-2 has many features that make it highly infectious including its glycoproteins, rapid entry through furin cleavage and TMPRSS2 (2–4), and suppression of host translation through Nsp1 (5–7). In response to the COVID-19 pandemic, the scientific community rallied to create new therapeutics and evaluate the effectiveness of any strategies that had been previously developed.

**Figure 1:**
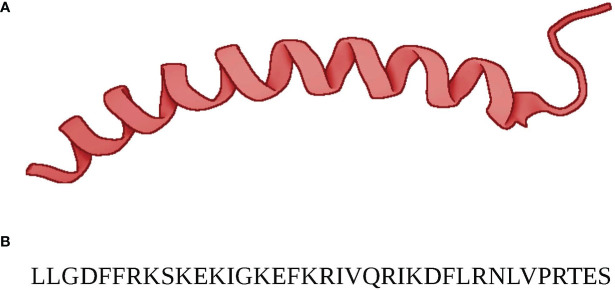
**(A)** The structure of (13)C,(15)N-labeled LL-37 determined by three-dimensional triple-resonance NMR spectroscopy for LL-37 in complex with micelles. (9) LL-37’s alpha-helical secondary structure is evident. **(B)** The 37 amino acid sequence of LL-37. At physiological pH LL-37 has a resulting net charge of +6.

In humans, the cathelicidin antimicrobial peptide (*CAMP*) gene encodes the pro-protein hCAP-18. Proteinase 3-mediated extracellular cleavage processes hCAP18 into the active 37 amino acid peptide, LL-37 (8). LL-37 is an amphipathic alpha-helical peptide that carries a positive charge of +6 at physiological pH (structure and chemical sequence are displayed in **Figure 1**). Many different cell types including barrier epithelial cells, macrophages, and neutrophils express this pro-protein and peptide throughout the body (10). The vitamin D pathway primarily regulates the *CAMP* gene (11–14). Recognition of bacterial or viral pathogens by Toll-like receptors (TLRs) activates cells to metabolize 25-hydroxyvitamin D [25(OH)D] to the active 1α,25-dihydroxyvitamin D [1,25(OH)_2_D]. This active form of vitamin D, binds to the vitamin D receptor (VDR), thus inducing the *CAMP* gene and numerous other vitamin D target genes involved in the immune response (14). Monocytes, macrophages and lung epithelial cells upregulate LL-37 expression *via* TLR-mediated activation of the vitamin D pathway (14–16).

LL-37 inhibits the propagation of SARS-CoV-2 through a direct mechanism. *In silico* docking studies have shown that LL-37 binds directly with the angiotensin converting enzyme-2 (ACE-2) binding domain that is critical to SARS-CoV-2 entry to host cells (17–19). These results were corroborated by *in vitro* and *in vivo* experimentation that found LL-37 not only blocks the receptor-binding domain (RBD) of SARS-CoV-2, but also cloaks the ACE-2 receptor preventing pseudovirion infection in cell culture and after intranasal application of LL-37 in mice (20). LL-37 also combats other viruses, such as influenza, rhinovirus, and respiratory syncytial virus, by causing disruption of viral membranes (21–24).

LL-37 functions as an antiviral and antibacterial peptide by inhibiting early steps in the viral replication cycle and perforating cytoplasmic bacterial membranes (25–27). In addition to penetrating bacterial membranes, LL-37 prevents biofilm formation and enhances bacterial phagocytosis (28, 29). LL-37 also kills *Candida albicans*, most effectively as a cleavage fragment RK-31 (30). Covid patients in the ICU for more than a few days commonly suffer from co-infections, and often with resistant species of bacteria and fungi, worsening prognosis (31–33).

In addition to its direct antimicrobial function, LL-37 modulates immune response and influences inflammation, cell proliferation and migration, wound healing, angiogenesis and the release of cytokines and histamine (34). Furthermore, recent studies indicate LL-37 plays an important role in neutrophil NETosis, which in turn can affect the formation and clearance of microthrombi (35–37). In effective NETosis, neutrophils respond to inflammatory stimuli by migrating to the infected tissue and decondensing their nuclear and mitochondrial DNA lined with granule proteins that incapacitate pathogens (NETs), followed by plasma rupture and NET release. These released NETs can trap the pathogens, which are subsequently cleared by DNase 1 and macrophages (38, 39). In COVID-19 patients, we see over accumulated NETs and that healthy neutrophils are more likely to engage in NETosis when prompted with SARS-CoV-2 patient serum (40). We hypothesis that LL-37 is important in both regulating the activation and clearance of NETs. These multiple functions are potentially highly relevant to SARS-CoV-2 infection and ameliorating COVID-19 symptoms and suggest LL-37 could function as a powerful therapeutic agent.

Studies from around the world investigating the correlation between health markers and COVID-19 severity have found statistically significant differences in serum 25-hydroxyvitamin D [25(OH)D] levels between patients having more and less severe COVID-19 outcomes, but others claim no correlation between serum 25-hydroxyvitamin D [25(OH)D] levels and infection or death rates (41–46) and many others discuss the therapeutic effects of vitamin D_3_ supplementation, referencing upregulation of cathelicidin gene expression and its antiviral capacity as a key component of the therapeutic and prophylactic power of vitamin D_3_ (44, 45, 47).

The effects of LL-37 are not limited to its ability to inhibit viral replication and infection. In this paper, we offer hypotheses rooted in previously published work to discuss the potential therapeutic and prophylactic uses of LL-37 as an effective tool in ameliorating COVID-19 pathology and reducing severe effects of COVID-19 infections. In addition, we describe strategies to regulate its expression with small molecules to achieve these goals.

## NETosis and Thrombosis

The lethality of SARS-CoV-2 is often attributed to its ability to induce thrombosis. Autopsies of COVID-19 patients reveal thrombosis in many of the narrow vessels as the primary cause of death. In one study involving autopsies of 10 COVID-19 patients, thrombosis and microangiopathy in the small vessels and capillaries led to an associated haemorrhage that significantly contributed to death. The lungs were found to have entangled neutrophils entrapped in fibrin and platelets forming thrombi in alveolar capillaries (38). Another study involving autopsies of 11 COVID-19 patients found thrombosis in the pulmonary arteries of all patients. This thrombosis was associated with heart attacks in eight of the patients and bronchopneumonia in six of the patients (48). A meta-analysis of 341 autopsies of COVID-19 patients bolstered the finding of thrombi in microvessels of the lungs, and additionally noted alveolar damage resulting in hyaline membrane formation (49). A study of blood from COVID-19 patients found that NET clearance was diminished and that these NETs instigate inflammation through interactions with anti-NET antibodies and macrophages. They found complications of NETs to include induction of alveolar cell apoptosis, mucus plugs, and capilaritis (50). These thrombi and observed alveolar damage develop from a complex interplay of inflammation and dysregulation of homeostasis.

## Vasoconstriction and Vasopermeability

One potential factor in this interplay is vasoconstriction in the lungs and other parts of the body due to the infection of pericytes by SARS-CoV-2. The binding domain affinity of SARS-CoV-2 for ACE-2 prevents the conversion of angiotensin II to angiotensin-(1-7), instigating constrictive behavior in pericytes. This effect has been observed in the brain of a Syrian golden hamster, which has an ACE2 sequence similar to human ACE2, and is hypothesized to occur in the pericytes of the heart and the kidney (51). Dysregulation of pericytes by the S protein of SARS-Cov-2 was also observed in pericytes sourced from human myocardial tissue (52). Constrictive behavior in pericytes present in the lungs may contribute to pulmonary thrombosis in COVID-19 patients by the mechanism discussed above, or through another interaction with inflammation pathways. Pericytes are associated with hypertension in vessels of the lungs of humans and rat models and increase in proliferation preceding hypertension after interaction with the inflammatory cytokine interleukin-6 (IL-6) (53). This cytokine is correlated with more severe COVID-19 cases (54).

## Role of NETs in COVID-19 Thrombosis

NETs are nuclear and mitochondrial DNA strands expelled from neutrophils through a process called NETosis. These strands form the backbone of weblike complexes studded with various peptides and proteins such as histones, lactoferrin, myeloperoxidase, neutrophil elastase, High Mobility Group Box 1, and LL-37 (39). The resulting NET complexes can bind and destroy infected cells, viruses, bacteria, and other pathogens around the neutrophils by exposing them to these peptides and proteins (39).

Beyond protecting against infectious agents, NETs can also have negative impacts on physiology by promoting tissue damage and thrombogenesis. These effects have previously been observed *in vitro* in neutrophils responding to endothelial damage in *Escherichia coli* infections (55). Toll-like receptor 4 (TLR4), which is present on the surface of platelets and activated by lipopolysaccharides (LPS) secreted during bacterial infection, encourages binding to neutrophils and promotes NETosis events in pulmonary capillaries (55). NETs can also adhere to platelets and form thrombi, and the histone proteins in NETs are sufficient to nucleate thrombi that matches those of deep vein thrombosis based on extracellular DNA presence and histone concentration in thrombi, as shown in a baboon animal model (36). The SARS-CoV-2 virus directly induces NETosis in healthy neutrophils isolated from patient serum, which demonstrates that COVID-19 patients face increased neutrophil NETosis rates as a direct result of the infection (40). This formation of thrombus scaffolds associated with increased NET production is compounded with increased thrombotic risk due to activation of the complement cascade/coagulation system by SARS-CoV-2 (56). Neutrophils from COVID-19 patients have been found to carry tissue factor (TF), an integral membrane protein involved in blood coagulation, and express elevated TF that is found within NETs (57).

The combination of vasoconstriction, nucleation of thrombi by SARS-CoV-2 induced NETs, and activation of the cascade/coagulation system is important to the pathology of COVID-19. Investigations into the histopathology of COVID-19 patients found vascular occlusions caused by NETs occurring in lung, kidney and liver tissue, and these occlusions disrupted circulation and induced endothelial damage (58 **)**. In lung specimens from patients with influenza and SARS-CoV-2 infection, researchers found neutrophil recruitment, NETosis, and subsequent immunothrombosis to be typical for severe COVID-19, but less prominent in influenza pneumonia (59). In Germany, a study of COVID-19 patients and healthy controls noted a significantly increase in NET markers in the plasma of COVID-19 patients (59, 60). Epithelial damage due to neutrophils activated by SARS-CoV-2 caused apoptosis of the A549 epithelial cell line, and this apoptosis was larger in magnitude than what was observed in neutrophils that were not activated by SARS-CoV-2. The neutrophils activated by SARS-CoV-2 released more NETs and were more cytotoxic than cells not activated by SARS-CoV-2 (61). NETs can also contribute to cytokine storm, a potentially fatal overstimulation of inflammation responses, through the stimulation of amongst others IL-6 (62). The foregoing factors—thrombosis, contribution to cytokine storm, and the impact of the granule proteins released by neutrophil congregations on endothelial cells— explain why increased levels of neutrophils are such a strong clinical indicator of severe COVID-19 outcomes (63). Consistent with this, in one recent analysis of a potentially fatal large vessel thrombus in a 28-year-old woman with COVID-19 infection, investigators ruled out all other instigators of thrombosis except infection, and they found the thrombus to have high neutrophil counts and a predominance of platelets (64).

The impact of age and other comorbidities on NETosis may explain the observed association with COVID-19. Researchers have identified chronic low-grade sterile inflammation, i.e. not caused by a pathogen, in elderly populations. High baseline serum concentrations of C reactive protein (CRP), IL-6, IL-8 and other cytokines characterize this inflammation (65). SARS-CoV-2 infection also appears to increase the production of IL-6 and IL-8, since higher levels of these cytokines are associated with more severe disease pathophysiology in COVID-19 patients (54). Amplification of these cytokines has also been observed in epithelial cells infected by previous coronaviruses, specifically SARS-CoV (66, 67). IL-8 chemoattracts neutrophils and T cells (68). IL-6 helps differentiate CD4+ T-cells, triggers the release of platelets from bone marrow, and reduces serological iron in response to lesions (69). Elevated IL-6 and IL-8 levels in the proximity of infected epithelial cells may lead to an increased concentration of neutrophils and platelets, and may thus result in thrombi instigated by the NET-nucleated thrombi interactions discussed above.

Based on the foregoing, we argue that NETs are highly relevant to COVID-19 pathology. Since neutrophil NETs are not observed to accumulate in extracellular regions in healthy tissues, physiological processes must exist for their removal after they have performed their immunological function. Although these processes are still not fully understood, some aspects of NET removal, and the cells that recycle them, have been elucidated. Current literature implicates deoxyribonuclease 1 (DNase 1), an enzyme that allows for the cleavage and modification of DNA, as an essential tool in NET removal. DNase helps with the degradation of the cellular and mitochondrial DNA that comprises the backbones of NETs. Curiously, while DNase can remove extracellular DNA, it failed to remove the majority of NET components adhered to the glycoproteins and glycosaminoglycans covering the endothelium (glycocalyx) which caused liver damage in a mice MRSA sepsis model (70). Moreover, simple upregulation of DNAse is insufficient to achieve NET removal, since DNAse by itself does not completely degrade NETs (71). Indeed, upregulating DNAse 1 could be detrimental, since NET fragments can boost certain bacterial coinfections such as Haemophilus influenzae (72, 73). Further elucidation of the mechanisms by which NET removal is achieved is critical to the development of procedures for effectively reducing NETosis in COVID-19 patients, and underscore the need for a greater understanding of the processes by which NET proteins that attach to the endothelium can reduce host tissue damage.

After NET processing, NET removal is achieved by macrophages through phagocytosis. Macrophages in the M2 state process the NETs and induce a pro-inflammatory response by releasing tumor necrosis factor-alpha (TNF-α), interferon gamma (IFN-γ), chemokine ligand 8 (CXCL8), chemokine ligand 10 (CXCL10/IP-10), chemokine ligand 12 (CXCL12/SDF-1) and clear extracellular DNA (74). Macrophages in the M1 state spew their own external DNA in a PAD-4 dependent manner during the initial interaction with NETosis, then utilize caspase activated DNase 1 to process the extDNA, and thereby clear extracellular DNA (extDNA) in roughly 24 hours. During the late phase this happens through a non-inflammatory mechanism (74). PAD4 catalyzes the conversion of protein-bound arginine into citrulline, resulting in a loss of positive charge thereby affecting chromatin structure. This distinction between macrophage states is particularly important for COVID-19. Indeed, it has been argued that macrophage activation and dysfunction is the key driver of COVID-19, and macrophage chemokine signatures indicate they are in the M1 state (74, 75). The issue of whether extracellular DNA from M1 macrophages can nucleate thrombi is important for determining major instigators of thrombosis in COVID-19 patients. The foregoing two-step process may represent an incomplete picture of NET clearance.

## LL-37’s Direct and Immunomodulatory Effects on the Clearance of NETs

Although clearance of NETs requires LL-37, the specific mechanisms involved are unclear. Removal of all the proteins attached to the DNA and histone backbone of the NETs abrogates phagocytosis by macrophages. Incubating these “naked” NETs with LL-37 restored *in vitro* phagocytosis of NETs, thus showing the importance of LL-37 in this process (35).

The existing literature reveals potentially relevant roles of LL-37 in NET clearance. LL-37 facilitates the *in-vitro* endocytosis of extDNA by dendritic cells through a process of DNA aggregation and condensation (76). LL-37 participates in the binding (through electrostatic forces), condensation, and uptake of extDNA in *in vitro* studies involving bacterial lysis and mammalian cell responses (77–79). The highly positive net charge of LL-37 (+6 at physiologically relevant ranges of pH) promotes interaction with DNA. Here, it is notable that DNA and LL-37 are both helical molecules, therefore, in some ways; they are structurally similar to each other. Their opposite charges allow them to interact *via* attractive electrostatic forces, a property allowing DNA condensation with LL-37 and promoting efficient phagocytosis by macrophages (70 **)**. We hypothesize that LL-37 aids clearance of NETs released by neutrophils and extDNA of M1 macrophages in COVID-19 patients, by binding to extDNA and condensing it into denser assemblies that activated macrophages can more effectively phagocytose. This may explain how administration of LL-37 helped clear histone-DNA complexes released from NETs in a murine sepsis model (80). Macrophages themselves can release LL-37 to facilitate this process further.

LL-37 also interacts with macrophages in other ways. It may serve as a signaling molecule for macrophages in the clearance of NETs. LL-37 regulates autophagy by macrophages *in-vitro* by activating transcription of autophagy-related genes (81). LL-37 also neutralizes LPS-mediated activation of macrophages and drastically reduces their production of TNF-α and IL-6 (82, 83). In some pathologies such as sepsis, LL-37 modulates many of the same cytokines seen in COVID-19 infections. LL-37 immunomodulation reduces levels of IL-6, TNF-α, IL-1β, and macrophage pyroptosis in sepsis-induced mice (84). In addition, LL-37 inhibits IL-6 production in macrophages treated with IFN-γ by inhibiting the p65 NF-κB signaling pathway (85). IL-6 is of particular concern because it has been identified as one of the most prominent cytokines in severe COVID-19 infections (54). Further investigation of LL-37 as a therapeutic to minimize macrophage dysfunction and the cytokine production associated with Macrophage Activation Syndrome (MAS) is warranted (86).

**Figure 2:**
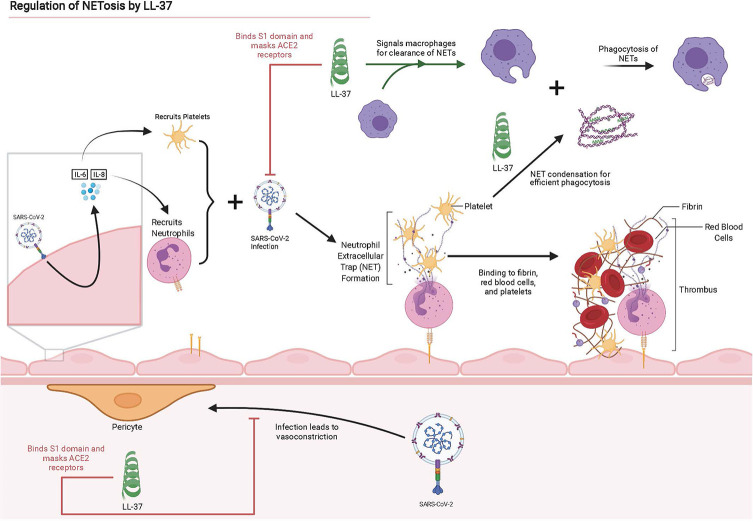
Graphical summary of interactions between SARS-CoV-2 and lung epithelium, instigating NET production and the disruption or antagonistic behavior of LL-37 for these interactions. Adapted from “The Propagation of Immunothrombosis by Leukocytes and Platelets”, by BioRender.com (2021). Retrieved from https://app.biorender.com/biorender-templates.

In addition to regulating macrophage activation, preincubation of influenza A virus (IAV) with LL-37 was shown to reduce expression of inflammatory cytokine IL-8 by neutrophils; IL-8 elevation is associated with more severe COVID-19 (54, 87). We hypothesize LL-37 is critical to the amelioration of COVID-19 and its sequelae through the condensation of neutrophilic DNA released in NETs, prevention of cytokine storm, and the signaling of macrophages to clear NETs as shown in **Figure 2**.

The power of citrullination is relevant to this discussion. Air pollution increases the citrullination of proteins and the formation of autoantibodies to these citrullinated proteins (88). This correlates with increased inhibition of NET clearance and defective NETs by a few mechanisms. The enzyme PAD4 catalyzes citrullination and decondensation of chromatin-releasing DNA and histones, which are expelled as NETs (89). As noted above, PAD4 facilitates extDNA release in macrophages (74). In addition, PAD4 can citrullinate LL-37, alter its antiviral activity, and increase the immune response by epithelial cells (90). In addition to air pollution, PAD4 is overexpressed in neutrophils from diabetic patients, making such neutrophils more likely to undergo NETosis and lose antiviral activity due to LL-37 citrullination. This modification also reduces the wound healing impact of LL-37 in instigating neovascularization and angiogenesis (91).

Another important consideration is that LL-37 helps induce and stabilize NETs. LL-37 is critical in the formation of NETs *in vitro*, which makes it a potential target for inhibiting the expulsion of NETs (37). It has also been found to increase NET production in response to IAV pre-incubated with LL-37, although research into its effects when introduced post-infection are undetermined (87). In one study, phorbol myristate acetate (PMA) was used to incite NET release in cultures of neutrophils. In some cultures, NET production doubled when treated with 10 μM of LL-37. In studies performed by Neumann et al. using parallel assays with a random LL-37 fragment library, a positive correlation was observed between NET induction and the hydrophobicity of LL-37 fragments, thus suggesting that hydrophobicity plays a role in the induction of NETs (37). Of course, one should critically analyze studies utilizing PMA for NET induction because differences in inflammation signatures from pathogens versus PMA induction exist.

LL-37 is essential to NET survival and persistence once deployed, because LL-37 inclusion within NET structures protects neutrophil DNA from cleavage by bacterial nucleases, including Staphylococcus aureus and Streptococcus pneumoniae (92). In this study Neumann et al., again, used parallel assays with a random LL-37 fragment library and found that cationicity was a critical factor to the function of LL-37 in protecting neutrophil DNA from cleavage by bacterial nucleases (92). While it is unclear whether LL-37 prevents degradation by human nucleases, it has been found that LL-37, and cationic antimicrobial peptides in general, help to stabilize neutrophil-derived DNA and NETs against bacterial nuclease degradation.

Another concern of LL-37 is a possible contribution to extracellular DNA concentrations. Previous studies have found that LL-37 induces macrophages toward M1 differentiation which we have already seen produce extracellular DNA similar to that seen in NETs since both are released *via* PAD4 dependent mechanisms (74, 93). If this extracellular DNA nucleates thrombi such as NETs or contributes to non-productive inflammation, then experiments to determine the ability of LL-37 to aid in phagocytosis of extracellular DNA as NETs should be weighed against the ability of LL-37 to stimulate extracellular DNA release from macrophages. The ability of LL-37 to reduce macrophage IL-6 production should also be weighed against its impact on IL-6 production in epithelial cells. Previous studies raise the potential concern that introduction of LL-37 to bronchial epithelial cells may increase expression of IL-6 (94). The study was performed in epithelial cells that were not introduced to pathogens and research into whether LL-37 increases IL-6 production in epithelial cells that have been infected should be done. In some applications, this concern should be taken into consideration or could be managed with drugs that interrupt the NF-κB signaling pathway that induces increased expression of IL-6 (94).

## Diabetes and COVID-19

Diabetes and hyperglycemia are significant comorbidities of COVID-19. Diabetic patients in a report from China were shown to have higher inflammatory serum markers and D-Dimer levels, which are linked to higher mortality in COVID-19 (95, 96). When comparing diabetic COVID-19 patients with non-diabetic patients the mortality risks increase with 1.9 odds ratio (OR), and risk of severe COVID-19 with 2.75 OR (97). A meta-analysis of 16 observational studies found that the OR of mortality among hyperglycemic patients relative to non-hyperglycemic patients was 3.45 and 2.08 for severe COVID-19 (98). The marked increases in COVID-19 severity and mortality associated with diabetes and hyperglycemia make them important areas of investigation to reducing the impacts of COVID-19.

Diabetes influences the host response to viruses in many ways. Notably, neutrophils from both type 1 and type 2 diabetic patients are primed to undergo NETosis, possibly due to an upregulation of PAD4, and wound healing is significantly delayed due to their presence (91). In addition to being primed to deploy NETs, neutrophils in diabetic mice were less likely to undergo apoptosis and clearance by macrophages. This leads to elevated levels of inflammatory cytokines such as TNF-α (99). These findings are consistent with clinical data, which shows patients with Type 2 Diabetes (T2D) have higher neutrophil-to-lymphocyte ratios and more severe COVID-19 outcomes than their non-diabetic counterparts (100). T2D has also been associated with higher calcium levels throughout the body (101, 102). This free calcium can play an important function in the regulation of NETosis events. Neutrophils isolated from human blood and stimulated by LPS and IL-8 show an increase in intracellular calcium. Moreover, treatment of neutrophils isolated from human blood with calcium ionophores promotes NET release (103–105). Hyperglycemia, which is related to diabetes (particularly T2D), is another major instigator of NETosis.

Hyperglycemia leads to activation of the polyol pathway, which enhances the formation of advanced glycation products, promotes the formation of reactive oxygen species that contribute to inflammation, and effectively reduces neutrophil opsonophagocytosis (106). Infection of β-cell islets by SARS-CoV-2 increases MAPK signaling, promotes β-cell apoptosis and exacerbates hyperglycemia (107–109). Hyperglycemia also “leads to greater MAP kinase signaling, NF-κB activity, and production of cytokines such as IL-6” (106). This general engagement of inflammatory cytokines is also associated with an increase in macrovascular complications (106). Another mechanism explaining the increase in macrovascular complications involves hyperglycemia and insulin resistance, which results in reduced intracellular Mg^2+^ (110). Magnesium deficiency increases the production of cytokines such as IL-1β, IL-6, TNF-α, vascular cell adhesion molecule-1, and plasminogen activator inhibitor-1 (111). IL-6 and TNF-α instigate neutrophil recruitment and activation (112). These results are consistent with the finding that hyperglycemia increases the release of NETs and circulating markers of NETosis (113). Lower serum magnesium is also associated with increased thrombotic risk and slowed fibrinolysis, which may contribute to, or stem from, NET-platelet thrombi (114). Hyperglycemia, and its associated hypertension, also, has a major impact on pericytes, causing vessels to weaken and form aneurysms in the case of diabetic retinopathy. This phenomenon may aid in explaining the increased severity of COVID-19 in diabetic patients (115).

Hyperglycemia causes significant disruptions in the renin–angiotensin–aldosterone system (RAAS). SARS-CoV-2 also disrupts the RAAS by increasing bradykinin levels through the downregulation of ACE, which clears bradykinin (116). As bradykinin accumulates, it increases the permeability of the local vasculature facilitating the recruitment of neutrophils, which instigates inflammation responses by releasing cytokines and perpetuating the cytokine storm (117–119). This explanation is consistent with the observation that polymorphonuclear leukocyte (PMN) infiltration in pulmonary capillaries and neutrophilic mucositis is observed in lung autopsies obtained from COVID-19 patients (120). This increase in vasopermeability and recruitment of neutrophils leads us to neutrophil extracellular traps (NETs), the next and arguably most important part of this complex inflammatory response interplay.

## LL-37 and Diabetes

In an *in vivo* murine model of Type 1 Diabetes (T1D), cathelicidin-related antimicrobial peptide (CRAMP) is expressed in insulin producing β-cell islets. CAMP/LL-37 served as a stimulator of pancreatic β-cell Ca^2+^ release and promoted the subsequent release of insulin or glucagon. CAMP/LL-37 treatment also stimulated β-cell neogenesis and enhanced the upregulation of potentially beneficial gut microbes in murine models (121). In addition, CRAMP/LL-37 modulated the inflammatory profile of pancreatic macrophages near β-cell islets in a dose-dependent manner (122). Reductions in the expression of cytokines such as TNF-α and IL-12 have been observed in macrophages from diabetic mice treated with LL-37 (122). Thus, LL-37 may be useful in addressing MAS that appears to be an essential driver of COVID-19 pathology (86) and of recently emerging cases of T1D induced by COVID-19 (123). These findings suggest that LL-37 upregulation may be instrumental in controlling blood sugar levels and to the healthy survival and growth of insulin producing cells. It may also be important in addressing the complications of COVID-19 associated with diabetes and hyperglycemia.

**Figure 3:**
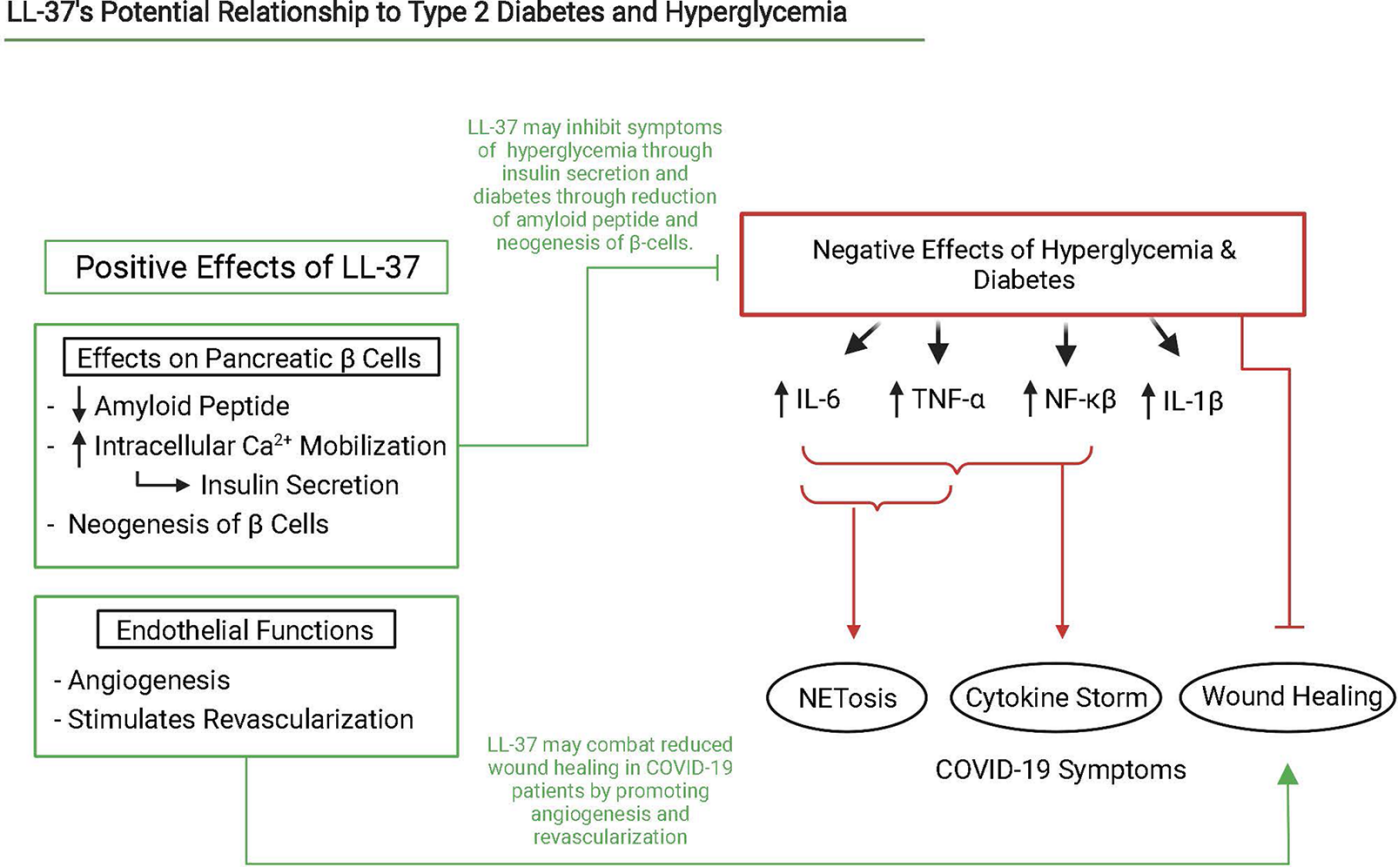
Interaction scheme of LL-37, NETosis, hyperglycemia, and diabetes that lead to severe COVID-19 symptoms. Relevant interactions are cited below. Created with BioRender.com.

In patients with T2D, NETs were found to contain LL-37, but its antibacterial abilities were found to be abrogated (124). Previous studies have shown that citrullination of LL-37 by PAD4 causes it to lose its antibacterial capacity, ability to promote clearance of extracellular DNA by dendritic cells, and even increases inflammatory responses in cells by abrogating some of LL-37’s immunomodulatory effects (125–128). We were unable to find literature investigating the impact of citrullination on LL-37’s antiviral capacity and recommend investigation into the field. It is known that T2D patients often overexpress PAD4, produce more NETs than non-diabetic reduced NET clearance, and mice models of T2D have increased difficulty in clearing NETs leading to decreased wound healing abilities (91). Patients treated with clarithromycin experience increased LL-37 load on NETs which enhanced wound healing and antibacterial and antiviral activity (124). We hypothesize that upregulated LL-37 can help control blood sugar levels, aid in combating diabetes, and act in a positive feedback loop to prevent citrullination and preserve its antimicrobial, immunomodulatory, and NET clearance activity (**Figure 3**).

In murine models, LL-37 improves wound healing in diabetic mice through its antimicrobial properties, while also directly activating endothelial cells in carrying out angiogenesis and neovascularization in repairing wounds (82). We hypothesize this is a function for addressing endothelial damage resulting from neutrophils and NETs responding to SARS-CoV-2 infections. LL-37 also prevents islet amyloid polypeptide (IAPP) self-assembly and subsequent β-cell damage *in vitro* (129).

## Neurological Symptoms of COVID-19

Observed neurological consequences of COVID-19 include chronic fatigue, confusion, dizziness, seizures, visual deficits, encephalopathy, encephalitis, loss of smell and taste, Guillan-Barre Syndrome, and more (130, 131). In the brain, cerebrovascular consequences of COVID-19 such as ischemic stroke and intracerebral hemorrhage can also lead to neurological complication and even death (132). In one case, a COVID-19 patient had acute necrotizing encephalopathy marked with the presence of SARS-CoV-2 RNA, astrocytic activation markers, neuronal injury markers, and more in the cerebral spinal fluid, ultimately leading to them in a coma (133).

There are many potential mechanisms by which COVID-19 can induce these neurological symptoms. In addition to thrombotic risk, NETs also pose potential harm to the central nervous system. It was found that virally activated neutrophils and hypothesized specifically that their NETs and reactive oxygen species (ROS) cause demyelination of the central nervous system in mice infected with a neurotropic coronavirus (134). SARS-CoV-2 activated neutrophils, which have been shown to produce more NETs than control neutrophils, may produce NETs and ROS that contribute to the cognitive dysfunction referred to as “brain fog” that COVID-19 patients experience (40). Knowledge of the ability of NETs to serve as scaffolds for thrombi make them a possible instigator of ischemic stroke seen as well (36, 58, 135 **)**.

Neurological complications may also result from infection of astrocytes in the brain by SARS-CoV-2. SARS-CoV-2 has been found to enter the CNS of rhesus monkeys through the olfactory route (136). Once in the CNS, they can trigger inflammatory sequences and dysfunction of surrounding cells. At least one study preprint found astrocytes are disproportionately infected by SARS-CoV-2 and express cell stress signals such as ARCN1 (137). We also saw increased astrocytic activation markers in the autopsy of the patient with acute necrotizing encephalopathy (133). Another preprint study analyzing infection by SARS-CoV-2 in macaques found the formation of Lewy bodies (138). These plaques, which are predominantly composed of alpha-synuclein, are associated with the pathology of Parkinson’s disease and have been hypothesized to derive from a viral etiology including influenza A, norovirus and others (139–141). In a preprint, data from autopsies corroborated the presence of SARS-CoV-2 in all lobes of the brains of COVID-19 patients although there was a lack of inflammation (142).

A high frequency of anti-neuronal autoantibodies in COVID-19 patients could also contribute to cognitive dysfunction (143). The infection of pericytes inducing their dysfunction, and possibly, their function in effectively regulating the blood-brain barrier (BBB) raises concern. Pericytes prevent vessel degeneration and BBB disruption, and act as phagocytes by performing pinocytosis (115). One *in vivo* preprint study of SARS-CoV-2 infection of pericytes revealed vasoconstriction in the BBB (51).

## LL-37 and SARS-CoV-2 in the Nervous System

**Figure 4:**
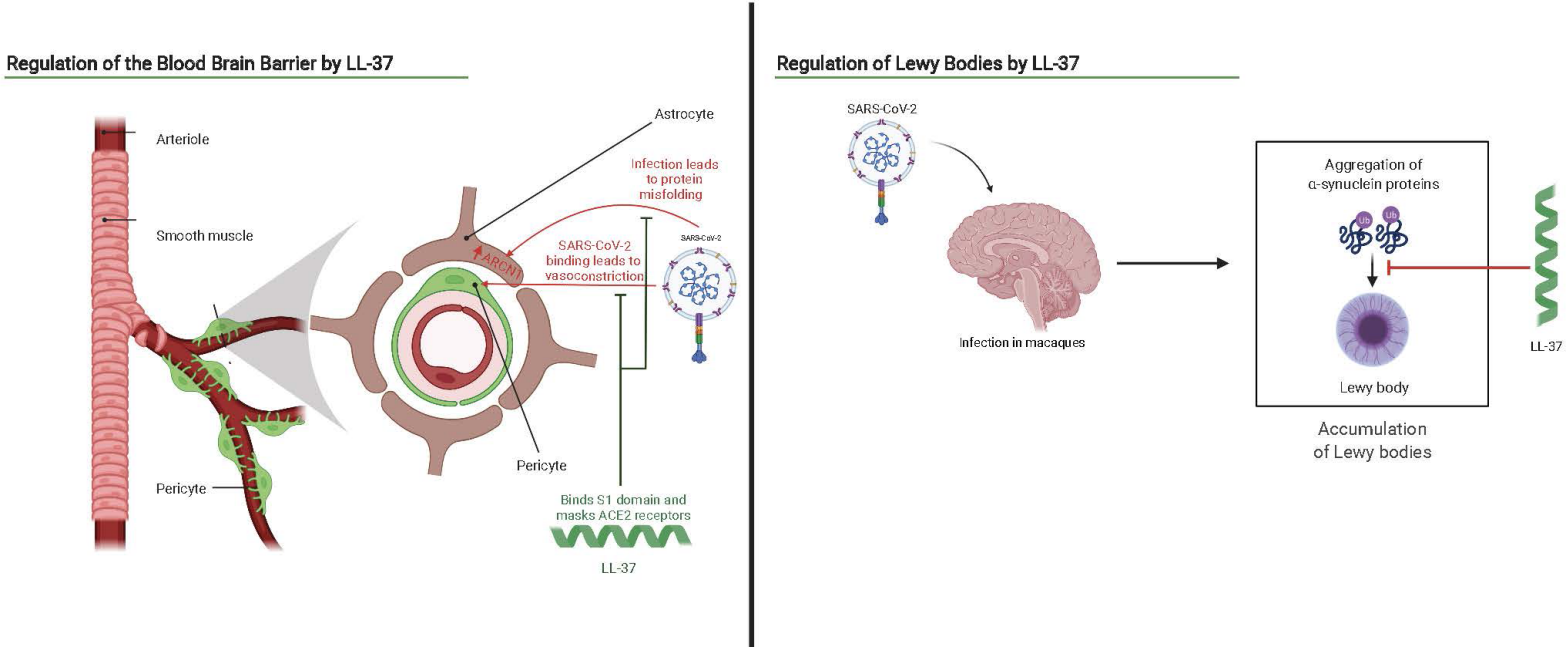
Interaction scheme of SARS-CoV-2 and the brain overlaid with potential antagonistic or therapeutic benefits of LL-37. Adapted from “Brain Vascular System” and “Progression of Parkinson’s Disease by the Substantia Niagra”, by BioRender.com (2021). Retrieved from https://app.biorender.com/biorender-templates.

Since the macaque model of COVID-19 has shown elevated levels in the brain of alpha-synuclein plaques, the role of alpha-synuclein production (if any) in common brain-fog and the attendant possible long-term impacts of COVID-19 is of interest (138). Similarly, since the brain has some of the highest levels of LL-37 expression (144) and LL-37 has previously been shown to suppress alpha-synuclein amyloid formation in cell culture, the impact of cathelicidin induction in addressing these sequelae deserves further investigation (145). We hypothesize enhanced LL-37 expression may address some consequences of COVID-19 by inhibiting alpha-synuclein aggregation and oligomer-induced cell damage and preventing infection of astrocytes as shown in **Figure 4**. Alpha synuclein plaques are associated with progression of Parkinson’s disease and LL-37 may also serve as a mechanism to reduce progression of the disease in COVID-19 patients. Vitamin D_3_, an up regulator of LL-37, has been hinted as helpful in addressing Parkinson’s in COVID-19 patients due to its super-promoter activity of Nrf2-KEAP, which promotes protective antioxidant and Ca^2+^ production and may work in conjunction with the potentially protective effect of LL-37 (146).

LL-37 may also provide a role in preventing pericyte dysregulation and astrocyte dysfunction through binding the S1 domain of SARS-CoV-2 and cloaking ACE-2 receptors (20). CRAMP has been shown to have antimicrobial activity in astrocytes of mice models responding to bacterial supernatants, demonstrating the co-location of cathelidin and astrocytes in response to pathogens (147). LL-37 may also prepare astrocytes for infection by gearing them into a pro-inflammatory state with upregulated IL-1β and IL-6, but this inflammatory state must be reduced upon infection to prevent chronic inflammation and disease (144). Research into the impacts of LL-37 in various cell lines of the brain post infection should also be conducted as a dual effect of LL-37 as pro-inflammatory in early infection but anti-inflammatory in cells that have encountered a pathogen has been previously noted (148).

## Inducers of LL-37

1,25(OH)_2_D_3_ upregulation of LL-37 is a highly conserved pathway in humans and one of the major inducers of LL-37 in the body (149, 150). In addition to vitamin D, some short-chain fatty acids are able to induce the expression of the *CAMP* gene, such as butyrate (151). However, butyrate is undesirable as a therapeutic compound, due to its noxious odor. Phenylbutyrate is a highly effective substitute, since it also induces *CAMP* gene expression but does not have a disagreeable odor (152). The combination of 1,25(OH)_2_D_3_ and phenylbutyrate is synergistic in its ability to induce *CAMP* gene expression in humans, providing enhanced expression of LL-37 and antibacterial activity, relative to what is achieved with just one of the two compounds (153). In the United States and most European countries, phenylbutyrate is an approved drug for the treatment of Urea-Cycle Disorders in both adult and pediatric patients (154). It was shown that phenylbutyrate induction of the *CAMP* gene required the vitamin D receptor (155). The synergistic induction of the *CAMP* gene by the combination of vitamin D_3_ and phenylbutyrate holds potential as a novel adjunct therapy for bacterial infections, particularly tuberculosis. Based on a dosage study done in humans, the ideal dose for induction of cathelicidin to treat lung infection is 5000 IU vitamin D_3_ taken daily, plus 500 mg phenylbutyrate per dose taken twice per day (156–159). Phenylbutyrate also showed promise in a preclinical animal trial using rabbits for the treatment of enteropathogenic *E. coli*-induced diarrhea (160). Certain phenylbutyrate analogs also induce *CAMP* gene expression. These include, for example, α-methylhydrocinnamate (ST7) (152).

Other compounds also induce the expression of the *CAMP* gene, although many of these inducers operate *via* mechanisms that are not fully understood and are independent of the VDR. For instance, the compound curcumin induces *CAMP* gene expression by a VDR-independent mechanism (161). Additionally, compounds from the family of stilbenoids (in particular resveratrol or pterostilbene) also induce *CAMP* gene expression by a VDR-independent mechanism, which is also synergistic with 1,25(OH)_2_D_3_ (162). Resveratrol induces cathelicidin expression by a novel mechanism involving sphingosine-1-phosphate pathway signaling (163, 164). Genistein, a soy-derived isoflavanoid, also induces cathelicidin expression by a sphingosine-1-phosphate stimulation mechanism (165).

Additional inducers of *CAMP* gene expression continue to emerge. In 2016, a novel family of compounds called aroylated phenylenediamines was developed to potently induce expression of the *CAMP* gene. This family includes the compound Entinostat, which has proven efficacious in treating shigellosis and cholera in rabbit models (166–168). Polysaccharide extracts from *Vaccaria segetalis* seeds (VSP) upregulated CRAMP expression in treated mice and LL-37 expression in A498 cells (169).

In this respect, it is notable that *CAMP* gene expression is observed in subjects upon exposure to certain external stimuli. For example, exercise induces cathelicidin expression in mice, even after very short (10-minute) sessions (170). Indeed, LL-37 is expressed in sweat, and is localized to both the eccrine gland and sweat ductal epithelial cells (171). Cathelicidin was also strongly induced by exposure to UVB ultraviolet light (172).

## Clinical Trials Evaluating Effects of LL-37’s Effects on COVID-19

Careful consideration of the drugs used to treat COVID-19 reveals relationships to LL-37. Clarithromycin, which is identified above as a regulator of LL-37 concentration on the surface of NETs, is currently under investigation in clinical trials. Metformin, an AMPK activator, has been found to facilitate clearance of NETs (173), decrease the production of pro-inflammatory cytokines and nuclear factors (such as NF-κB) when bound to certain Toll-like-receptors (such as TLR4) (174), and increase insulin sensitivity to reduce hyperglycemia by downregulating NF-κB and TLR4 (175). LL-37 shows similar characteristics as it stimulates the P2X7 receptor which stimulates autophagy in macrophages, in combination with phenylbutyrate and AMPK signaling (176). LL-37 also reduces the expression of TLR4 in murine dendritic cells (177) and lessens TLR4 activation by LPS in J774 macrophages (178). No studies analyzing LL-37’s impact on virally stimulated macrophages were found in our search. TLR4 has been hypothesized to regulate the severity of COVID-19 by binding to SARS-CoV-2 and upregulating cell-surface expression of ACE-2 (179). LL-37 has also been found to modulate blood glucose level effectors and participate in NET clearance as discussed above. These studies suggest a possible clinical benefit in upregulating LL-37.

## Discussion

The therapeutic benefits of inducing LL-37 (**Table 1**) warrant investigation, not only as a tool for combating SARS-CoV-2 infection but also for applications to the ongoing discovery of longitudinal symptoms and known consequences of infection. The unexplored role of LL-37 in NET formation and clearance may prove to be of critical importance in preventing and ameliorating COVID-19-associated microthrombosis. Furthermore, the ability of LL-37 to instigate bronchial revascularization and angiogenesis has the potential to be critical for recovery. The ability of LL-37 to encourage insulin release and its role in the proper function of NETs in diabetic and hyperglycemic patients could provide another tool in the fight against SARS-CoV-2, since it addresses a major comorbidity of severe COVID-19. Lastly, LL-37 may disrupt some of the consequences of infection, such as alpha-synuclein plaque deposition in the brain, and IAPP plaque deposition in the pancreas associated with the development of insulin insensitivity. The immunomodulatory powers of LL-37 expression have been referenced by recent studies on the effects of vitamin D_3_ supplementation in modulating COVID-19 disease progression (44, 180). The vitamin D pathway directly induces LL-37 in humans, contributes to a myriad of other benefits to the immune system, and appears to reduce mortality rates by 80% in COVID-19 patients in the UK (181, 182). This relationship is relevant in the lungs, the tissue most impacted by the disease. Lung epithelial cells can change inactive vitamin D_3_ to its active form, which subsequently produces active LL-37 peptide locally (15, 183). Further investigations of the vitamin D_3_/LL-37 axis in relation to SARS-CoV-2 may be crucial to the creation of a widely accessible therapeutic strategy to combat infection and disease caused by this rapidly evolving virus.

**Table 1:** List of potential therapeutic effects of LL-37 against COVID-19 separated based on mechanism of action and system targeted.

*Mechanism*	*Aspect of Physiology*	*Symptom Addressed*	*Role of LL-37*
***Antiviral***	Vasculature	Infection of pericytes that leads to dysregulated constrictive behavior that can narrow blood vessels, increasing risk of thrombosis.	LL-37 directly binds to SARS-CoV-2 and inhibits infection of pericytes.
		SARS-CoV-2 downregulates ACE, thereby, accumulating Bradykinin and increasing vasopermeability to polymorphonuclear leukocytes (PMN), cytokine release, and neutrophilic mucositis.	LL-37 directly binds to SARS-CoV-2 preventing it from downregulating ACE thereby preventing accumulation of bradykinin.
	Neurological	Astrocytes are disproportionately infected by SARS-CoV-2 and their infection has been hypothesized as the source of the symptom “brain-fog” associated with COVID-19.	LL-37 directly binds to SARS-CoV-2 and can incapacitate the virus before it can infect astrocytes.
		Infection of pericytes impairs their ability to effectively regulate the blood-brain barrier (BBB). Pericytes prevent vessel degeneration and BBB disruption, and act as phagocytes by performing pinocytosis. One *in vivo* study of SARS-CoV-2 infection of pericytes revealed vasoconstriction in the BBB.	LL-37 directly binds to SARS-CoV-2 and inhibits infection of pericytes that leads to dysregulated constrictive behavior that can narrow blood vessels and affect the integrity of the BBB.
***Immuno-modulatory***	Vasculature	Thrombosis - Neutrophil extracellular traps (NETs) serve as scaffolds for fibrin and platelets to bind and form thrombi in COVID-19 patients. These thrombi are the cause of death in some patients. NETs also increase production of interleukin 6, which is associated with cytokine storm.	LL-37 has been proven necessary for NET removal and we hypothesize it does so through the condensation of the DNA in NETs and signaling macrophages for NET clearance.
	Metabolism	Hyperglycemia and diabetes are associated with more severe COVID-19 due to creation of reactive oxygen species, higher baseline inflammation, increased NET production, and reduced NET clearance.	LL-37 can combat hyperglycemia and prevent beta-islet cell incapacitation through promotion of Ca^2+^ mobilization that leads to insulin release, Beta-islet cell neogenesis, prevention of islet amyloid polypeptide self-assembly, and reducing inflammatory profiles of macrophages surrounding Beta-islet cells. LL-37 can also stimulate angiogenesis and neovascularization to support repair of endothelial damage due to early infection.
	Neurological	Virally activated neutrophils can cause demyelination of the CNS.	LL-37 reduces the inflammatory immune profile of neutrophils that have been stimulated by a pathogen.
		SARS-CoV-2 infection in a macaque model showed infection in the brain and production of Lewy bodies, collections of alpha-synuclein plaque associated with Parkinson’s disease.	LL-37 can inhibit alpha-synuclein aggregation and oligomer induced cell damage.

## Data Availability Statement

The original contributions presented in the study are included in the article/supplementary material. Further inquiries can be directed to the corresponding author.

## Author Contributions

KMA and JAF drafted the manuscript. JEN, EBD, JSL, MS, JDC, AFG, and AEB reviewed, discussed, and performed critical editing. All authors read and approved the final manuscript.

## Funding

We would like to thank the NIH for funding this work with a Pioneer Award to Annelise Barron, grant # 1DP1 OD029517-01. AB also acknowledges funding from Stanford University’s Discovery Innovation Fund; from the Cisco University Research Program Fund and the Silicon Valley Community Foundation, from Stephen Pearse, and from Dr. James J. Truchard and the Truchard Foundation. JEN was funded by grant NNF21OC0068675 from the Novo Nordisk Foundation and the Stanford Bio-X Program. JC is supported in part by funds provided by the Texas A&M University System.

## Conflict of Interest

The authors declare that the research was conducted in the absence of any commercial or financial relationships that could be construed as a potential conflict of interest.

## Publisher’s Note

All claims expressed in this article are solely those of the authors and do not necessarily represent those of their affiliated organizations, or those of the publisher, the editors and the reviewers. Any product that may be evaluated in this article, or claim that may be made by its manufacturer, is not guaranteed or endorsed by the publisher.
